# SARS-CoV-2 Genome Sequence from Morocco, Obtained Using Ion AmpliSeq Technology

**DOI:** 10.1128/MRA.00690-20

**Published:** 2020-07-30

**Authors:** Farah Jouali, Nabila Marchoudi, Fatima Zahra El Ansari, Yassine Kasmi, Mohamed Chenaoui, Aissam El Aliani, Nawfel Azami, Salma Loukman, Moulay Mustapha Ennaji, Rachid Benhida, Jamal Fekkak

**Affiliations:** a Molecular Biology Department, ANOUAL Laboratory, Casablanca, Morocco; b Biomedical Genomics and Oncogenetic Research Laboratory, Faculty of Sciences and Techniques, Tangier, Morocco; c Molecular Genetics Laboratory, Thünen Institute for Fisheries Ecology, Bremerhaven, Germany; d Research Center of Plant and Microbial Biotechnologies, Biodiversity, and Environment, Faculty of Sciences, Mohammed V University, Rabat, Morocco; e Laboratory of Virology, Microbiology, Quality, Biotechnologies/Eco-Toxicology, and Biodiversity, Faculty of Sciences and Techniques, Mohammedia, University Hassan II, Casablanca, Morocco; f Chemical and Biochemical Sciences Department, Mohammed IV Polytechnic University, Ben Guerir, Morocco; DOE Joint Genome Institute

## Abstract

This study describes a genome sequence of severe acute respiratory syndrome coronavirus 2 (SARS-CoV-2) sampled from a male patient with SARS-CoV-2 who was likely infected in Casablanca, Morocco.

## ANNOUNCEMENT

In December 2019, a cluster of patients with severe acute respiratory symptoms was identified in Wuhan, China. Later, it was declared that a new emerging severe acute respiratory coronavirus was behind these infections (1). This new virus is classified within the *Coronaviridae* family, specifically the *Betacoronavirus* genus. This novel coronavirus’s high transmission capacity from human to human (2) led to its fast expansion worldwide, causing a disease outbreak. In March 2020, the World Health Organization (WHO) declared the beginning of a pandemic caused by severe acute respiratory syndrome coronavirus 2 (SARS-CoV-2) and referred to as coronavirus disease 2019 (COVID-19) (3). Since then, many research projects have been conducted to better understand its replication and pathogenicity to develop prevention, diagnostic testing, and treatment strategies (4).

As a contribution to the global efforts to track and trace the ongoing coronavirus pandemic, here, we present the sequence of a SARS-CoV-2 genome that was obtained from a mildly symptomatic Moroccan patient. All participants gave written informed consent to participate in the study. This study was approved by the Ethics Committee for Research of the University Hassan II.

Nasopharyngeal swab specimens were collected from five patients using swabs with a synthetic tip, and the tip was immediately inserted into a sterile tube containing 1 to 3 ml of viral transport medium (LQ Amies). RNA was automatically extracted using the Maxwell 16 viral LEV kit. A total of 300 μl of viral transport medium was added to 300 μl of lysis buffer and 30 μl of proteinase K, and the mixture was incubated for 10 min at 56°C. After lysis of the sample, the lysate was transferred to a Maxwell 16 LEV cartridge.

The five patients were found to be PCR positive for SARS-CoV-2 after a real-time reverse transcriptase PCR (RT-PCR) assay using a Da An gene kit (Sun Yat-sen University, China). The sample with the highest viral load was selected for next-generation sequencing (NGS) analysis (threshold value [*C_T_*], 16 for the N gene and 21 for the ORF1ab gene).

The SARS-CoV-2 viral genome sequencing was performed manually using the Ion AmpliSeq technology and the Ion Torrent personal genome machine (PGM). Briefly, the extracted viral RNA was quantified using Qubit assays (Life Technologies, USA) to ensure that we were using an adequate RNA concentration (1 to 10 ng). cDNA was synthesized with the SuperScript VILO reverse transcriptase kit (Invitrogen, USA). The libraries were prepared using the Ion AmpliSeq library kit version 2.0 (Life Technologies) and Ion AmpliSeq SARS-CoV-2 research assay panel according to the manufacturer’s instructions. This panel consists of two primer pools that generate 237 amplicons covering the entire SARS-CoV-2 genome and 5 human expression controls. The prepared library underwent template preparation with the Ion OneTouch 2 and Ion OneTouch ES systems using the Ion OneTouch Hi-Q view OT2 kit version 2 (Life Technologies) according to the manufacturer’s instructions.

The enriched templates were then loaded onto an Ion 316 chip for semiconductor sequencing on the Ion PGM machine using the Ion PGM Hi-Q view sequencing kit according to the manufacturer’s instructions.

The fastq file was uploaded from the Torrent Suite software version 5.0.5 and then processed for quality assessment using the FastQC version 0.11.5 program. Sequence trimming was performed with the Trimmomatic version 0.32 program. The resulting reads were then mapped to the complete genome of the SARS-CoV-2 Wuhan-Hu-1 isolate (GenBank accession number MN908947.3) using both the Bowtie 2 version 2.3.3.1 and Burrows-Wheeler Aligner (BWA) version bwa-0.5.9 programs. Our final consensus sequence is 29,838 bp long with a 37.98% GC content. We generated a total of 977,015 reads with a mean coverage depth of 600× and an average length of 169 bp. The genome sequence was compared to the complete genome of the SARS-CoV-2 Wuhan-Hu-1 isolate using the Betacoronavirus BLAST tool and showed 99.8% similarity.

**FIG 1:**
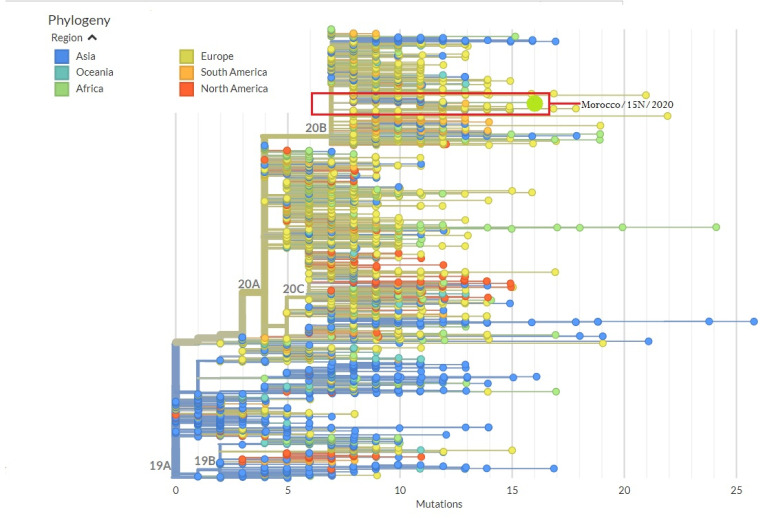
Phylogenetic tree of the SARS-CoV-2 genome from Morocco and the related coronaviruses. The *x* axis represents the number of mutations from the Wuhan strain (GenBank accession number MN908947.3). The large green circle represents the position of our sequence, Morocco/15N/2020. According to the phylogenetic tree, the ancestral genotypes closest to ours include sequences from North America, Africa, Asia, and Europe. The figure was rendered using Nextstrain (https://nextstrain.org/ncov/global).

The phylogenetic analysis of this genome sequence was performed using the Nextstrain tool enabled by data from the Global Initiative on Sharing All Influenza Data (GISAID) (Fig. 1). Our results revealed that this sequence belongs to clade B11 with high similarity to those reported in Florida under GenBank accession number MT276329.1, in Israel under accession number MT276598.1, and in France under accession number MT470156.1.

Comparing our genome with others from Morocco shows that current circulating strains in Morocco came from different countries with a local evolution. More studies should be conducted to clarify virus spread in our country as well as its origin.

### Data availability.

This sequence has been deposited in the GISAID emerging coronavirus SARS-CoV-2 platform under identifier EPI_ISL_458150, in the GenBank database under the accession number MT568645.1, and in the Sequence Read Archive (SRA) database under the accession number SRR12119018. The Betacoronavirus BLAST URL is https://blast.ncbi.nlm.nih.gov/Blast.cgi?PAGE_TYPE=BlastSearch&BLAST_SPEC=Betacoronavirus.
